# Analysis of tuberculosis prevalence surveys: new guidance on best-practice methods

**DOI:** 10.1186/1742-7622-10-10

**Published:** 2013-09-28

**Authors:** Sian Floyd, Charalambos Sismanidis, Norio Yamada, Rhian Daniel, Jaime Lagahid, Fulvia Mecatti, Rosalind Vianzon, Emily Bloss, Edine Tiemersma, Ikushi Onozaki, Philippe Glaziou, Katherine Floyd

**Affiliations:** 1 London School of Hygiene & Tropical Medicine London, UK; 2 World Health Organization Geneva, Switzerland; 3 Research Institute of Tuberculosis (RIT), Tokyo, Japan; 4 Department of Health, Manila, Philippines; 5 University of Milano-Bicocca, Milan, Italy; 6 Centers for Disease Control and Prevention (CDC), 1600 Clifton Rd, MS E-10, Atlanta, GA 30333, USA; 7 KNCV, The Hague, The Netherlands

## Abstract

**Background:**

An unprecedented number of nationwide tuberculosis (TB) prevalence surveys will be implemented between 2010 and 2015, to better estimate the burden of disease caused by TB and assess whether global targets for TB control set for 2015 are achieved. It is crucial that results are analysed using best-practice methods.

**Objective:**

To provide new theoretical and practical guidance on best-practice methods for the analysis of TB prevalence surveys, including analyses at the individual as well as cluster level and correction for biases arising from missing data.

**Analytic methods:**

TB prevalence surveys have a cluster sample survey design; typically 50-100 clusters are selected, with 400-1000 eligible individuals in each cluster. The strategy recommended by the World Health Organization (WHO) for diagnosing pulmonary TB in a nationwide survey is symptom and chest X-ray screening, followed by smear microscopy and culture examinations for those with an abnormal X-ray and/or TB symptoms. Three possible methods of analysis are described and explained. Method 1 is restricted to participants, and individuals with missing data on smear and/or culture results are excluded. Method 2 includes all eligible individuals irrespective of participation, through multiple missing value imputation. Method 3 is restricted to participants, with multiple missing value imputation for individuals with missing smear and/or culture results, and inverse probability weighting to represent all eligible individuals. The results for each method are then compared and illustrated using data from the 2007 national TB prevalence survey in the Philippines. Simulation studies are used to investigate the performance of each method.

**Key findings:**

A cluster-level analysis, and Methods 1 and 2, gave similar prevalence estimates (660 per 100,000 aged ≥ 10 years old), with a higher estimate using Method 3 (680 per 100,000). Simulation studies for each of 4 plausible scenarios show that Method 3 performs best, with Method 1 systematically underestimating TB prevalence by around 10%.

**Conclusion:**

Both cluster-level and individual-level analyses should be conducted, and individual-level analyses should be conducted both with and without multiple missing value imputation. Method 3 is the safest approach to correct the bias introduced by missing data and provides the single best estimate of TB prevalence at the population level.

## Background

National population-based surveys of the prevalence of pulmonary tuberculosis (TB) disease in adults can be used to measure the burden of disease caused by TB, to measure trends in this burden when repeat surveys are performed and to understand why people with TB have not been detected or diagnosed by national TB control programmes (NTPs). Surveys are of greatest relevance in countries with a high burden of TB in which surveillance data capture much less than 100% of cases. Global targets for reductions in disease burden set for 2015 include halving prevalence rates compared with their level in 1990; the other targets are that mortality rates should be halved between 1990 and 2015, and that TB incidence should be falling by 2015 [1].

**Figure 1:**
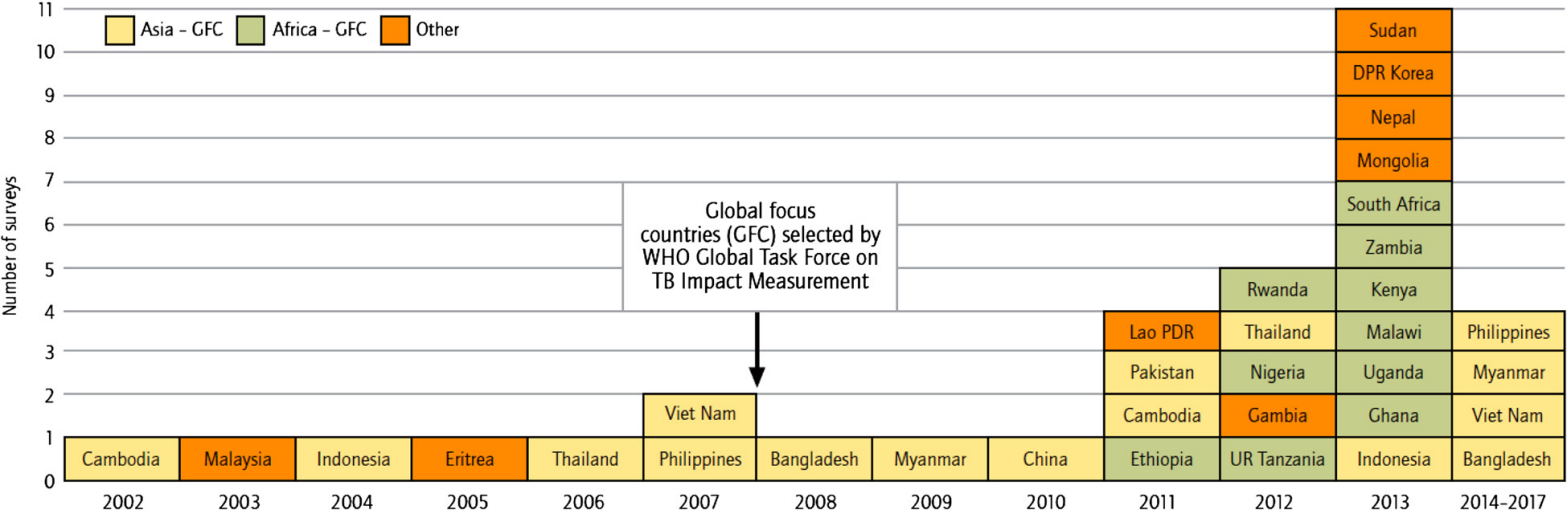
**Global progress with nationwide prevalence surveys of TB disease.** Global progress in implementing field operations of nationwide surveys of the prevalence of TB disease, actual (2002–2012) and expected (2013–2017).

The Global Task Force on TB Impact Measurement is hosted by the World Health Organization (WHO) with a mandate to ensure the best-possible assessment of whether 2015 global targets for reductions in TB disease burden are achieved [2]. The Task Force has strongly recommended national TB prevalence surveys in 22 global focus countries in the years leading up to 2015 [3,4]. Since 2008, there has been an unprecedented increase in the number of countries either implementing or planning to implement nationwide surveys. Between 2009 and 2015, approximately 23 countries - including 20 of the global focus countries - are expected to implement a survey, compared with a total of 7 countries in the period 2002–2007 (Figure 1). Only four countries, all in Asia, implemented surveys between 1990 and 2001. The global investment in prevalence surveys will amount to around US$ 50 million between 2010 and 2015. Analysis of results using best-practice methods is crucial.

TB prevalence surveys have a cluster sample survey design, in which groups of individuals are sampled, with clusters selected at random from an area sampling frame with probability proportional to size (PPS). While the classic method of using each survey cluster as the unit of analysis has been carefully and thoroughly described for a TB prevalence survey [5,6], methods to implement an individual-level analysis, in which each eligible adult enumerated in the survey is the unit of analysis, have not. An individual-level analysis is valuable because it enables adjustment for differences between participants and non-participants and multiple imputation of missing data, while simultaneously allowing for clustering in the sampling design. Missing data in TB prevalence surveys can be observed in both the outcome (TB case or not) but also other covariates, for example due to non-participation of eligible individuals, unavailability of screening or diagnostic results due to human error, and loss of specimens at laboratories for reasons such as contamination. A prevalence estimate based on only individuals with complete data will be biased, except under the strong assumption that those with and without full information have the same prevalence of TB. Methods that incorporate missing value imputation are thus important for two reasons: to obtain a more valid estimate of pulmonary TB prevalence, and to assess the bias of simpler analytical approaches [7,8]. Moreover, while participation rates in recent surveys in Asia have been very high, the rates achieved in other surveys from 2012 onwards may be lower; accounting for missing data will become essential for production of robust results.

Findings from national TB prevalence surveys completed in 2007 in the Philippines and Viet Nam have been published [9,10]. Other national surveys have either not followed the screening strategy now recommended by WHO [11,12], or the results have been disseminated in a survey report but not in a scientific journal. The analysis of the Philippines survey attempted to account for missing data using within-cluster mean imputation, stratified on age and sex, but did not include individual-level analysis. The analysis of the Vietnam survey used an individual-level analysis but did not formally account for missing data on smear and culture results, or age and sex differences between participants and non-participants.

**Figure 2:**
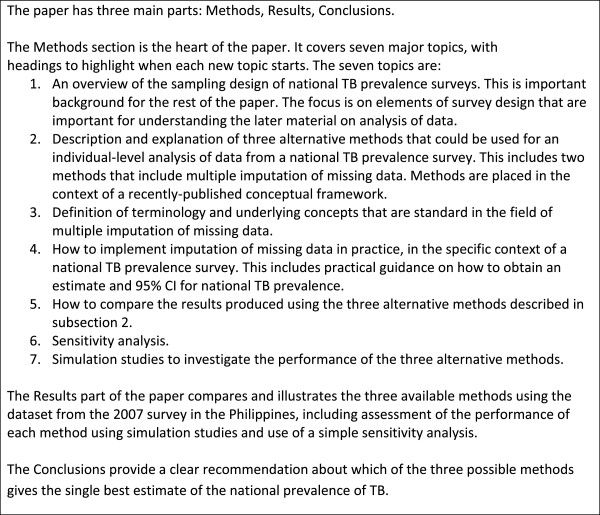
Paper outline.

This paper (outlined in Figure 2) provides new theoretical and practical guidance on best-practice methods for analysis of data from a TB prevalence survey, notably methods for individual-level analyses that account for the cluster sample survey design and that allow correction for biases due to missing data. Methods are described and explained, and then illustrated and compared using data from the 2007 survey in the Philippines. We draw on material previously developed in 2010 by the authors in a WHO handbook [4] but provide much more explanation of the underlying principles and methods required to implement multiple imputation of missing data. This includes guidance based on insights gained in 2011 and 2012 through the analysis of prevalence surveys conducted in Myanmar (2010) [13], Ethiopia (2010/11) [14], and Cambodia (2010/11) [15]. We also place the analytical methods within a new conceptual framework [16,17].

## Methods

### Survey design and summary of key data: an overview

For on-going and future TB prevalence surveys, the eligible population is defined as individuals aged ≥15 years old who were already resident in the selected cluster at the time of the survey team’s first pre-survey visit [4]. Individuals <15 years old are excluded, because of the difficulties in diagnosing pulmonary TB in children. Cluster size is recommended to be between 400 and 1000 eligible individuals, with the target cluster size constant within a particular survey [4]. Typically 50–100 clusters are selected, depending on the total sample size required. Sample size is calculated with the aim of estimating the population prevalence of pulmonary TB among eligible individuals with 20-25% relative precision [4]. In most surveys that have already been completed, participation of eligible individuals has been of the order of 85%-95%, with typically lower participation in urban areas. Most surveys use stratification, to ensure that the number of clusters allocated to each stratum is in proportion to the population in that stratum. For example, the Philippines 2007 survey had three strata (urban, rural, and the capital city) [9].

**Figure 3:**
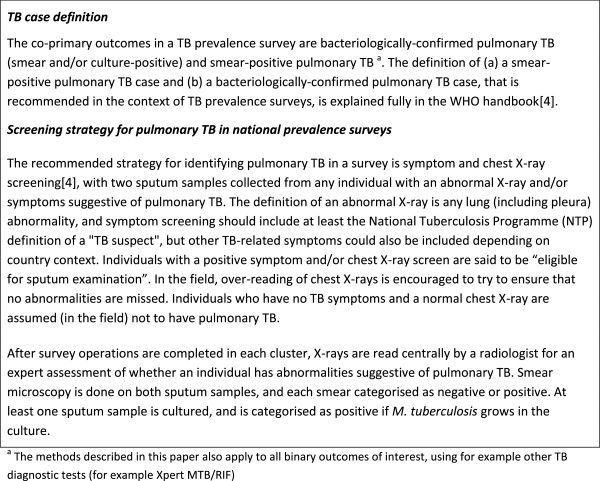
TB case definition, and screening strategy for pulmonary TB.

There are 2 co-primary outcomes in a TB prevalence survey: (1) smear-positive pulmonary TB and (2) bacteriologically-confirmed pulmonary TB (smear-positive and/or culture-positive). The TB case definition, and the screening strategy used to identify pulmonary TB, in a national-level prevalence survey are summarised in Figure 3.

**Figure 4:**
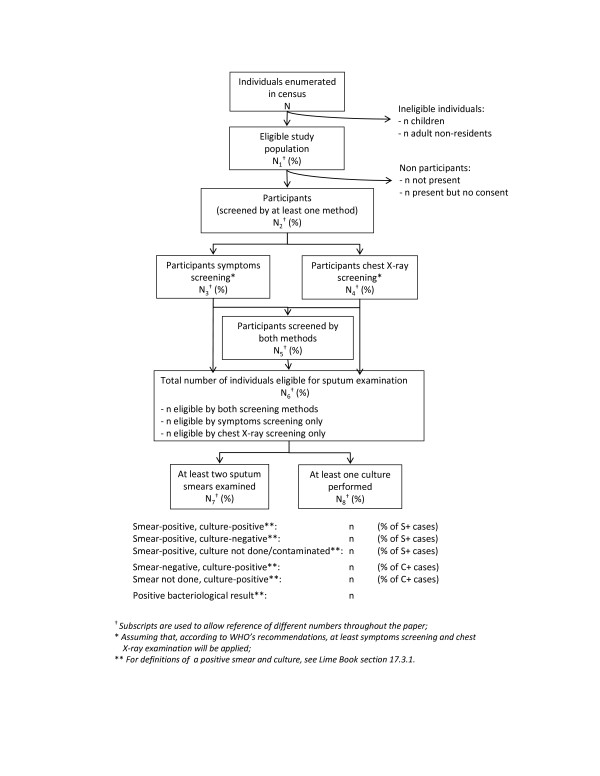
**Survey participant flow.** Schematic of numbers of participants screened for TB in the prevalence survey according to survey protocol.

The number of individuals who were enumerated, were eligible to participate, and who participated at various stages of the survey should be summarised, for example as depicted in Figure 4.

Before analysis of the two co-primary outcomes is done, it is essential to describe the completeness and internal consistency of the “core” data i.e. the data that it is essential to collect in all TB prevalence surveys. This is covered in detail in the WHO handbook [4].

### Individual-level analysis of pulmonary TB prevalence: description and explanation of three alternative methods

The two outcomes of smear-positive pulmonary TB and bacteriologically-confirmed pulmonary TB should be analysed separately. Here, we illustrate methods for an individual-level analysis using the outcome of bacteriologically-confirmed pulmonary TB, which we will refer to hereafter as pulmonary TB. It should be noted that the analytical approach would also be the same for other outcomes that are binary (yes or no), for example TB diagnosed using the recently endorsed molecular test Xpert MTB/RIF [18].

Individual-level analyses of pulmonary TB prevalence are performed using logistic regression, in which the log odds, i.e. $\log\left(\frac{\pi_{\mathrm{ij}}}{1-\pi_{\mathrm{ij}}}\right)$ is modelled, where *π*_*ij*_ is the probability of individual *i* in cluster *j* being a prevalent pulmonary TB case. The simplest model that can be fitted is $\alpha =\log\left(\frac{\pi_{\mathrm{ij}}}{1-\pi_{\mathrm{ij}}}\right)$, in which case α is estimated as $\alpha =\log\left(\frac{p}{1-p}\right)$, where *p* is the observed overall proportion of study participants with pulmonary TB. Correspondingly $p=\frac{\exp\left(\alpha \right)}{1+\exp\left(\alpha \right)}.$ Logistic regression is used because the outcome is binary i.e. for each individual there is a probability that they have pulmonary TB at the time of the cross-sectional survey (in the generalised linear models framework, the logistic link function is the “natural link function”). The most crucial characteristic of such analyses is that they take into account the clustering of individuals: if this is not done, the calculated 95% confidence interval (CI) for true pulmonary TB prevalence will have less than the nominal 95% coverage, due to underestimation of the standard error of the prevalence estimate.

Two types of logistic regression model are recommended for the analysis of a TB prevalence survey, both of which allow for the clustering in the sampling design. These are: (1) logistic regression, with robust standard errors calculated from observed between-cluster variability and (2) random-effects logistic regression, in which a parameter for between-cluster variation in pulmonary TB prevalence is included in the probability model.

Random-effects logistic regression models may be preferred for quantifying the association between risk factors and pulmonary TB prevalence, because they provide a full probability model for the data including the between-cluster variability in true TB prevalence. However, the estimation process used in these models produces a “shrunken” point estimate of the overall nationwide pulmonary TB prevalence that is too low because it is calculated as a geometric, and not arithmetic, mean of the observed cluster-specific prevalence values. Therefore, robust standard error logistic regression models, which are “population-average” models within a generalised estimating equations framework, are preferred for the overall estimation of nationwide pulmonary TB prevalence.

**Figure 5:**
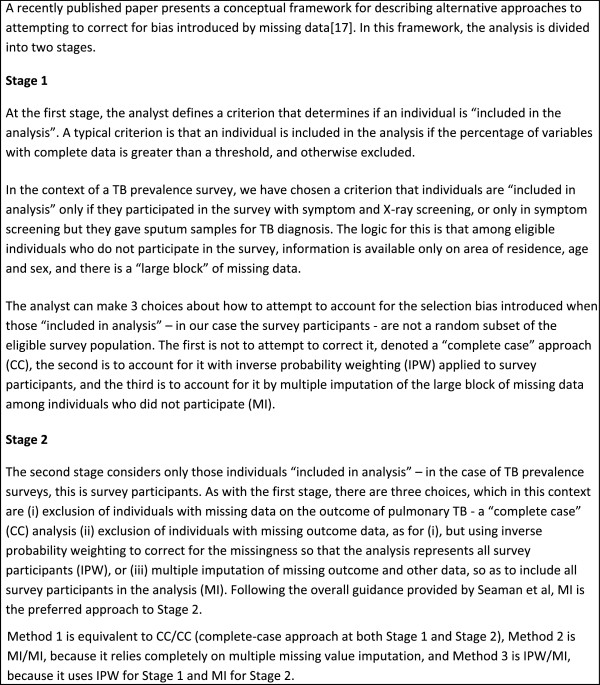
Methods 1-3, placed within a conceptual framework for analytical methods that attempt to correct for bias introduced by missing data.

To estimate overall pulmonary TB prevalence, it is recommended to use 3 methods of analysis in total, one of which does not account for missing data and two of which attempt to correct for bias due to missing data. In Figure 5, we place all three methods within the framework set out in a recent paper that considers the combination of inverse probability weighting (IPW) and multiple imputation (MI), with the analysis divided into two stages [17]. Method 1 is equivalent to CC/CC (complete-case approach for both Stage 1 and Stage 2), Method 2 is MI/MI, to indicate it relies completely on multiple missing value imputation, and Method 3 is IPW/MI, to indicate it combines inverse probability weighting (for Stage 1) with multiple imputation (for Stage 2).

#### Method 1 (complete-case or CC/CC)

This method uses a logistic regression model with robust standard errors, no missing value imputation, and analysis is restricted to survey participants (=N_2_ in Figure 4), and also excludes individuals who were eligible for sputum examination but smear and/or culture results are missing. Individuals who were not eligible for sputum examination are assumed not to have pulmonary TB, unless their chest X-ray was later found to be suggestive of TB based on a reading at “central level” by an experienced radiologist – in which case they are also excluded from the analysis. The model does not account for variation in the number of individuals per cluster, or correlation among individuals in the same cluster, when estimating the point prevalence of pulmonary TB. Equal weight is given to each participating individual in the sample. However, the model does correct for clustering (by using the observed between-cluster variation) when estimating the 95% CI, and can control for stratification in the sampling design. This method corresponds to a classical individual-level analysis of a survey, in the case that one does not need to adjust for sampling weights. TB prevalence surveys are designed to be “self-weighted”, with each individual in the population having the same probability of selection into the sample [4] and thus the same “weight” in the analysis. Among *participants*, this method always *underestimates* true TB prevalence – because data on pulmonary TB are missing only among individuals who were eligible for sputum examination, who have a relatively higher probability of being a TB case compared with those not eligible. Differential participation in the survey by cluster, age group, and sex may either exacerbate or reduce this bias.

#### Method 2 (MI/MI)

This method uses a logistic regression model with robust standard errors, with missing value imputation for survey non-participants as well as participants, and includes all individuals who were eligible for the survey in the analysis (=N_1_ in Figure 4). Multiple missing value imputation (additional details below) is used for all individuals: a) without a field chest X-ray result and/or symptom screening – which includes all individuals who did not participate in the survey, b) with a field chest X-ray reading that the survey protocol stated should also be read at central level, but missing the central reading, c) eligible for sputum examination but whose status as a pulmonary TB case is unknown due to missing smear and/or culture results and d) ineligible for sputum examination, but with a central X-ray reading that was suggestive of TB, whose status as a pulmonary TB case is thus unknown. This method allows for both the clustering in the sampling design and the uncertainty introduced by imputation of missing values when estimating the 95% CI for the prevalence of pulmonary TB.

#### Method 3 (IPW/MI)

The third method is also a logistic regression model with robust standard errors, with missing value imputation done among the subset of survey participants who were eligible for sputum examination but for whom smear and/or culture results were missing, and inverse probability weighting applied to all survey participants. This method aims to represent the whole of the survey eligible population (=N_1_ in Figure 4), but the weights are applied only to individuals who participated in the survey. An individual is considered to have participated in the survey if they were screened by both chest X-ray and symptoms, or they refused or were exempted from X-ray screening but provided sputum samples for TB diagnosis (=N_5_ in Figure 4). Missing value imputation is used for individuals eligible for sputum examination (=N_6_ in Figure 4), plus individuals who were not eligible for sputum examination but whose chest X-ray was read as suggestive of TB at central level, for whom data on one or more of the central chest X-ray reading, symptom questions, and smear and/or culture results were not available. Inverse probability weighting is then used to correct for differentials in participation in the survey by age, sex, and cluster. This is considered the “safer” method compared with Method 2 because a smaller amount of missing data is imputed. This means that if the imputation model is miss-specified, the bias in the resulting estimates will be smaller.

### Missing value imputation: key concepts in the context of TB prevalence surveys

Three main types of missing data mechanism have been distinguished in the literature [7,8]; we explain them below in the context of data being missing for the primary outcome variable, prevalent pulmonary TB.

(i) *Missing completely at random (MCAR): no adjustment required*

Data are MCAR if the probability that an individual has missing data on the outcome, pulmonary TB, is NOT related to either a) the value of the outcome (that is, TB case yes or no) or b) an individual characteristic that is a risk factor for the outcome (for example age, sex, stratum, cluster, TB symptoms). In this case, analysis can be restricted to individuals who DO participate fully in the survey, and an unbiased estimate of the true overall prevalence of pulmonary TB in the population will be obtained. In other words, the (probabilistic) sampling design itself automatically allows for “completely at random” missing data.

(ii) *Missing at random (MAR): missing value imputation required*

In the context of a TB prevalence survey, data are MAR if two conditions are fulfilled. First, the probability that an individual has missing data for the outcome variable of pulmonary TB (yes or no) *is* related to individual characteristics such as age, sex, stratum, TB symptoms, and the field chest X-ray reading. Second, *within* groups of individuals who are the same for age, sex, stratum, TB symptoms, and field chest X-ray reading, the probability of data being missing on the outcome variable is *not* associated with its value (that is, pulmonary TB case yes or no).

If data are MAR, the observed prevalence of pulmonary TB can be used to predict TB (yes or no) for individuals for whom data are missing, provided this is done with stratification on at least an individual’s age, sex, area of residence, TB symptoms, and field chest X-ray reading. Having done this, an unbiased estimate of the true overall prevalence of pulmonary TB in the population can be obtained.

(iii) *Missing not at random (MNAR): missing value imputation and also sensitivity analysis required*

Data are MNAR if the probability of an individual having missing data on the outcome variable (that is, TB case yes or no) is different for individuals who have pulmonary TB compared with individuals who do not have pulmonary TB, even after post-stratification of individuals using characteristics that are known to be risk factors for pulmonary TB (such as area of residence, age, sex). If data are MNAR, it is not possible to correct the estimate of pulmonary TB prevalence simply by using missing value imputation based on the patterns in the observed data. Instead, a sensitivity analysis is required (see below), which is an area of on-going research [19].

The observed data themselves cannot be used to distinguish between MAR and MNAR. Missing value imputation is implemented under the assumption that data are MAR.

### Missing value imputation: recommended approach to implementation

#### Method 2

In a TB prevalence survey, it is usually the case (based on experience to date) that age, sex, stratum, and cluster are known for all (or almost all) eligible individuals, while there will be missing data on TB symptoms, field and central chest X-ray readings, smear and culture results, and the primary outcome of pulmonary TB.

It is essential to start by exploring the extent to which data are missing, in order to understand the possible biases that may result from an analysis that is restricted to survey participants and to choose imputation models that make the MAR assumption plausible. The following three variables should be summarized: the proportion of eligible individuals who participated in the symptom and chest X-ray screening; the proportion of those with two sputum samples among people eligible for sputum examination; and the proportion with smear and culture results from 0, 1 or 2 sputum samples. These summaries should be done overall, and be broken down by individual risk factors for pulmonary TB such as age group, sex and stratum – in order to know which individual characteristics are predictors of missingness.

Missing value imputation is done using regression models in a procedure called “imputation by chained equations”, and can be implemented using standard statistical software packages such as Stata, SAS, and R [20-22]. For example, in the statistical package Stata this is done using the *ice* (“imputation by chained equations”) command [23]. Additional file 1 explains, step-by-step, how the imputation is implemented to create a single imputed dataset. As recently set out in a paper that provides general guidance on the use of multiple imputation [16], key principles to observe when specifying the imputation model are: (1) it must include all explanatory variables to be investigated as risk factors at the analysis stage, and the outcome variable itself; (2) to make the MAR assumption plausible it “should include every variable that *both* predicts the incomplete variable *and* predicts whether the incomplete variable is missing”; (3) including variables that are predictors of the incomplete variable, whether or not they also predict missingness, will give better imputations; and (4) including variables that are predictors of missingness, whether or not there is statistical evidence they are predictors of the incomplete variable, helps to limit the potential for bias.

Our recommendation, following from this, is as follows. The outcome variable in a TB prevalence survey is pulmonary TB; sputum smear and culture results, the field and central chest X-ray reading, and TB symptoms are used in combination to define if an individual has pulmonary TB (see Additional file 1 for more detail). Thus all of these variables must be included in the imputation models. Individual characteristics that are established predictors of pulmonary TB (e.g. age, sex) and/or predictive of data being missing (e.g. age, sex, stratum) should be considered for inclusion in the imputation models, as illustrated in Additional file 1. The strongest predictors of pulmonary TB and/or missingness (age, sex, stratum) should *always* be included in the imputation models for TB symptoms, field X-ray reading, and smear and culture positivity. At the same time, the choice of additional predictors (e.g. smoking and alcohol consumption) may need to be limited so as to avoid severe collinearity, especially when imputing smear and culture results and the number of positive smear and culture results is small (though because imputation models are being used for predictive purposes, moderate collinearity is not problematic). Including cluster as an explanatory variable in the imputation model with smear positivity (yes or no) as the outcome variable is not recommended, because the number of individuals with a positive smear result is low relative to the number of clusters; this is true also for the imputation model with culture positivity (yes or no) as the outcome variable. For outcomes that are more common, such as abnormal chest X-ray result (yes or no), including cluster as an explanatory variable in the imputation model may be appropriate.

The process described in Additional file 1 is repeated to create, for example, 10–20 imputed datasets (hence the terminology “multiple” missing value imputation). The number of imputed datasets should be greater than or equal to the percentage of eligible individuals for whom data are missing [16]. To date, this percentage has been in the range 4-15% in TB prevalence surveys, and we recommend that at least 20 imputed datasets are created.

The overall prevalence of pulmonary TB is calculated for each imputed dataset. The national-level pulmonary TB prevalence estimate is then calculated as the average of the pulmonary TB prevalence values from each imputed dataset, with a 95% CI that takes into account both the sampling design and the uncertainty due to missing value imputation. In Stata, this can be done using the *mim* or *mi* commands [23].

#### Method 3

Multiple imputation is an efficient method for accounting for missing data, provided the imputation models are specified appropriately [8,16,24]. An alternative approach is to use a *combination* of multiple imputation (MI) and inverse probability weighting (IPW) [17]. With this approach, imputation is used to fill in missing values only among individuals who participated fully in the survey (N_5_ in Figure 4).

Survey participants can be divided into two groups, eligible or ineligible for sputum examination. Individuals who were ineligible for sputum examination are assumed not to have pulmonary TB, unless they had a normal field chest X-ray reading but an abnormal central chest X-ray reading. For those eligible for sputum examination (N_6_ in Figure 4, and additionally individuals with a normal field chest X-ray reading but abnormal central chest X-ray reading), multiple imputation is used to fill in missing data, in exactly the same way as described for Method 2 above (including using the same variables in the imputation models). Each of the imputed datasets is then combined with the data on individuals who were ineligible for sputum examination, to give (for example) 20 imputed datasets that include all individuals who participated fully in the survey.

For each imputed dataset, a point estimate and 95% CI for population pulmonary TB prevalence is then calculated, using logistic regression with robust standard errors and weights. Weights are calculated for each combination of cluster, age group, and sex. This is done by a) counting the number of eligible individuals in each combination of cluster, age group, and sex (N_ijk_, for cluster *i*, age group *j*, sex *k*) and b) counting the number of survey participants in each combination of cluster, age group, and sex (n_ijk_). The weight for each individual is then equal to N_ijk_ / n_ijk_, for the particular combination of cluster/age group/sex that they are in, with n_ijk_ / N_ijk_ being the probability that the sampled individual participates in the survey – hence the name “inverse probability weighting”. It is essential to include either the weights or the covariates that predict the weights in the imputation model [17]. We include age group, sex, and stratum (area of residence) in all imputation models. An average of the estimates of pulmonary TB prevalence from each of the imputed datasets is then calculated, together with a 95% CI. In Stata, this can be done using the *mim* and *svy* commands.

An advantage of using IPW combined with MI, rather than just MI, is that it is relatively simple and transparent to calculate the probability of survey participation by cluster, age group and sex, compared with adjusting for non-participation through the use of a multivariable imputation model [17,24]. However, an important assumption remains, which is that after post-stratifying on cluster, age, and sex, the prevalence of pulmonary TB is the same in survey participants and non-participants.

### Comparing results across Methods 1–3

If point estimates of pulmonary TB prevalence and their confidence intervals vary greatly among Methods 1–3, it is essential to try to understand the reasons for the differences and the results of the survey should be interpreted within these limitations. Method 1 introduces biases, as explained above, so it is not surprising if it provides a prevalence estimate that is different to the one obtained from Methods 2 and 3. If the prevalence estimates from Methods 2 and 3 are considerably different, this may be due to misspecification of the imputation models used in Method 2.

### Sensitivity analysis: a simple method

A simple way to implement a sensitivity analysis is to use as a starting point the imputed datasets that were created using Method 2.

For an “extreme” situation in which there are 0 pulmonary TB cases among non-participants, the prevalence of pulmonary TB is estimated simply as the observed number of pulmonary TB cases divided by the total eligible survey population. For an opposite “extreme” in which the risk of pulmonary TB is twice as high among non-participants as in participants (within sub-groups defined by stratum, age group, sex, and other variables included in the imputation model for pulmonary TB), the number of pulmonary TB cases among non-participants is estimated for each imputed dataset as 2t_i_, where t_i_ is the number of pulmonary TB cases that were imputed in the i^th^ imputed dataset. Then the overall pulmonary TB prevalence is calculated as the average of the 2t_i_ values, plus the number of pulmonary TB cases among survey participants, divided by the total eligible survey population.

### Simulation studies to assess the performance of Methods 1–3

Simulation studies were done for 4 plausible scenarios through which missing data could be generated in TB prevalence surveys. We explored missingness of data on the outcome of prevalent TB by age, sex, stratum and cluster. We chose these four variables on the basis that they are associated both with the outcome and the reason for missingness [16]. Across the 50 clusters in the 2007 Philippines survey, the minimum number of individuals aged ≥10 years old for whom data on all of age, sex, stratum (urban, rural, the capital city), cluster, field and chest X-ray reading, TB symptoms, and smear and culture results, were complete was 190. In order to create a dataset in which the number of individuals in each cluster was the same, all TB cases in each cluster and a random sample of non-TB cases were selected to create a dataset of 9500 individuals, i.e. 190 in each of 50 clusters. In this dataset, TB prevalence was 1263 per 100,000 (120/9500).

Missing values were then introduced into this dataset to create 1000 datasets with missing data on the field chest X-ray reading and TB symptoms, and smear and culture results, for each of the following 4 scenarios:

1. Differential participation by age group, sex, and stratum (n = 3), with overall participation approximately 90%; 15% of smear and culture results missing completely at random among individuals eligible for sputum examination; overall, 19% of eligible individuals with missing data on pulmonary TB.

2. Differential participation by age group, sex, and cluster (n = 50), with overall participation approximately 90%; 15% of smear and culture results missing completely at random among individuals eligible for sputum examination; overall, 20% of eligible individuals with missing data on pulmonary TB.

3. As for 2, but among individuals eligible for sputum examination, the probability of missing smear and culture results varied among the 3 strata; overall, 20% of eligible individuals with missing data on pulmonary TB.

4. As for 2, but among individuals eligible for sputum examination the probability of missing smear and culture results varied among the 50 clusters; overall, 20% of eligible individuals with missing data on pulmonary TB.

## Results

### An example analysis using the dataset from the 2007 survey in the Philippines

To illustrate the 3 methods of analysis outlined above, we use the 2007 national TB prevalence survey in the Philippines. In this example, the eligible survey population was individuals aged ≥10 years old, which is different from the current WHO recommendation for the survey population to consist of individuals aged ≥15 years old [4]. However, the analytical approach and presentation of results remain the same.

Overall, participation was high at 90% of eligible individuals, though it was higher in rural and urban areas than in the capital city, lower among 20–39 year olds than other age groups, and the age-pattern of survey participation differed between men and women (data not shown). Additional details about the survey are provided elsewhere [9].

### Comparison of results across Methods 1–3

**Figure 6:**
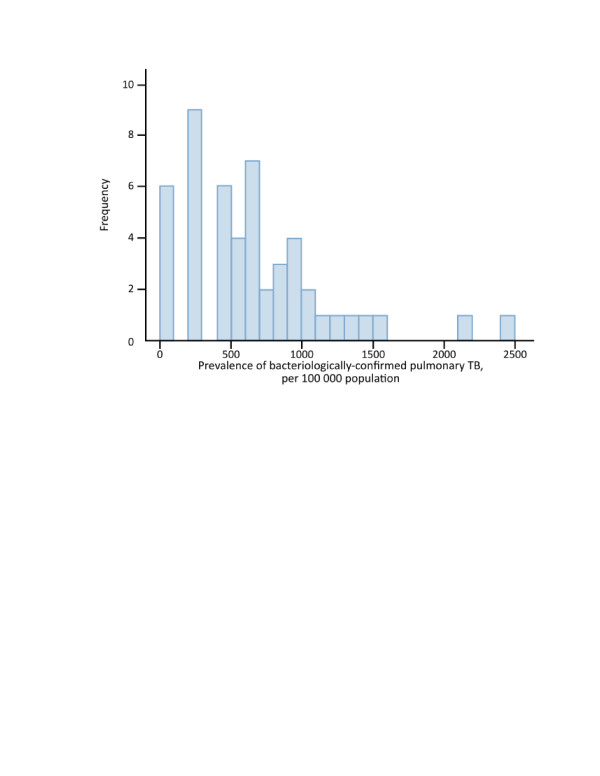
**Distribution of cluster-level prevalence of bacteriologically-confirmed pulmonary TB among 50 clusters, Philippines, 2007*.***

Results for the prevalence of pulmonary TB are summarised in Table 1, and the observed distribution of cluster-level pulmonary TB prevalence is shown in Figure 6. From the cluster-level analysis, the estimate of the prevalence of pulmonary TB is 663 per 100,000 population, with a 95% confidence interval of [516–810], with Method 1 giving an almost identical estimate and 95% confidence interval. Method 2 gives the same point estimate of pulmonary TB prevalence but with a slightly narrower confidence interval.

**Table 1:** Prevalence of pulmonary TB (per 100,000 population) in the Philippines 2007 national TB prevalence survey

**Prevalence (95% CI)**	**Cluster-level**	**Method 1**^**1**^	**Method 2**^**2**^	**Method 3**^**3**^
*Overall point prevalence*	663 (516–810)	660 (520–810)	660 (530–800)	680 (530–830)
*Point prevalence by stratum*^*4*^				
Metro Manila	671 (238–1105)	670 (100–1240)	640 (160–1120)	710 (100–1320)
Other urban	671 (421–921)	660 (470–860)	680 (500–860)	700 (490–910)
Rural	655 (447–863)	660 (450–870)	650 (460–850)	660 (440–870)
	**n / N (Prevalence, 95% CI)**			
*Overall crude*^5^*prevalence*	136/20 544 (660, 560–780)			
*Stratum crude*^5^*prevalence*				
Metro Manila	15/2253 (670, 370–1100)			
Other urban	50/7519 (660, 490–880)			
Rural	71/10,772 (660, 520–830)			

^1^Robust standard errors.

^2^Robust standard errors with missing value imputation.

^3^Robust standard errors with missing value imputation and inverse probability weighting.

^4^Stratum-specific estimates are calculated from an overall regression model including all clusters and all individuals, with stratum fitted as a fixed-effect in the model.

^5^Crude prevalence is calculated as the total number of individuals with a positive smear and/or culture result divided by the total number of individuals who have been screened for TB by chest X-ray and/or interview. Confidence interval for this estimate is calculated with exact binomial probability theory.

The point prevalence estimate of pulmonary TB from Method 3, combining multiple imputation with inverse probability weighting, is slightly higher than the estimates from Methods 1 and 2, at 680 per 100,000 and with a slightly wider confidence interval.

Among survey participants, multiple imputation of missing smear and culture results increases the estimate of the prevalence of pulmonary TB from 660 to 670 per 100,000. This is a relatively small increase, reflecting that among individuals eligible for sputum examination the proportion with missing data on smear and/or culture results was very low. Using inverse probability weighting to account for differentials in survey participation by cluster, age group, and sex increases the prevalence estimate from 670 to 680 per 100,000.

Overall, the cluster-level analysis and the results from each of Methods 1, 2, and 3 show that the best estimate of pulmonary TB prevalence is of the order of 660 – 680 per 100,000 population among individuals aged ≥10 years old, with the coverage of the 95% CIs ranging from 516 to 830 per 100,000 population.

### Sensitivity analysis

A sensitivity analysis in which pulmonary TB prevalence among non-participants ranges from 0 to being twice as high as among participants, gives a range of the point estimate of pulmonary TB prevalence from 595 to 731 per 100,000 population, compared with the estimate from Methods 1 and 2 of 660 per 100,000.

### Simulation studies to assess the performance of Methods 1–3: results

**Figure 7:**
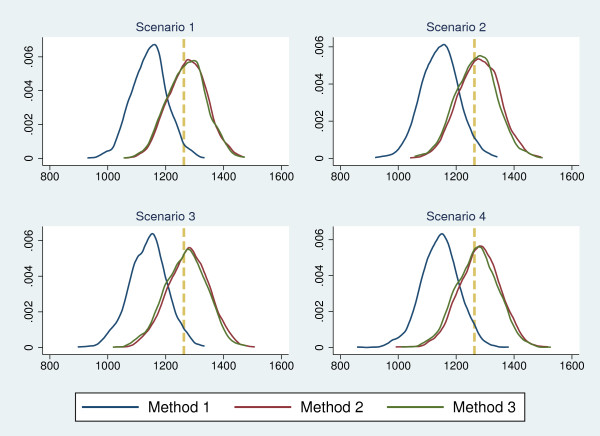
**Density plots of simulated data series.** Density plots of the distribution of prevalence estimates calculated from simulation study data series. Dashed vertical line represents the “true” level of prevalence.

For all of scenarios 1–4, we analysed each of the 1000 datasets using Methods 1, 2 and 3. For both Methods 2 and 3, 20 imputed datasets were created for each of the 1000 “starting” datasets. Simulation results showed that for all 4 scenarios, Method 1 underestimated TB prevalence by an average of approximately 9%, with prevalence estimates lower than the true value of 1263 per 100,000 for 97% of the Scenario 4 simulations. Method 2 overestimated TB prevalence by an average of around 1.5%, while Method 3 estimated TB prevalence to an average that was within 1% of the true value. Details of the results are summarised in Table 2 and Figure 7.

**Table 2:** Simulation study results, for 4 scenarios of how missing data could arise in a prevalence survey

	**Method 1**^**1**^		**Method 2**^**2**^		**Method 3**^**3**^	
	**Mean (SD)**^**4**^	**Relative bias (%)**^**5**^	**Mean (SD)**^**4**^	**Relative bias (%)**^**5**^	**Mean (SD)**^**4**^	**Relative bias (%)**^**5**^
*Scenario 1*	1143 (60.0)	−10	1276 (65.5)	1.0	1273 (66.0)	0.8
*Scenario 2*	1144 (64.0)	−9	1279 (70.4)	1.3	1270 (70.1)	0.6
*Scenario 3*	1139 (65.0)	−10	1278 (71.4)	1.2	1269 (71.8)	0.5
*Scenario 4*	1144 (64.8)	−9	1281 (71.2)	1.4	1272 (71.7)	0.7

^1^Robust standard errors.

^2^Robust standard errors with missing value imputation.

^3^Robust standard errors with missing value imputation and inverse probability weighting.

^4^Mean estimate of pulmonary TB prevalence (per 100,000 population aged ≥10 years old), over 1000 simulations, and standard deviation of the 1000 pulmonary TB prevalence estimates. The true value of TB prevalence in these data was 1263 per 100,000 population.

^5^The relative bias is defined as the percentage = (mean-true)/true. Negative values indicate under-, positive values over-, estimation of the true prevalence by the simulated series of data.

## Conclusions

We recommend that the method that uses the cluster as the unit of analysis should remain the first step in the analysis of a TB prevalence survey [6], as it is a simple method of analysis that has the advantage of being very transparent. It also requires a careful description of the variation in observed cluster-level pulmonary TB prevalence, which is an important feature of the data that should be described well and summarized graphically. However, it has exactly the same limitations in terms of bias as an individual “complete-case” analysis (Method 1). In our simulation studies, Method 1 underestimated TB prevalence by an average of about 9%. It is thus essential that a cluster-level analysis is followed by individual-level analyses, initially restricted to individuals for whom data on the primary outcome of pulmonary TB is complete (a “complete-case analysis”), and then extended through missing value imputation to include all eligible individuals.

Following a general recommendation [7], it is important to present both the complete-case analysis (Method 1) and an analysis that attempts to correct for bias introduced by missing data. Following recent work on using a combination of IPW and MI [17], it is also recommended to always compare the approach that uses multiple imputation for all eligible individuals (Method 2) with the more conservative approach that uses multiple imputation only among survey participants and uses IPW to account for differences between participants and non-participants (Method 3). Our simulation studies show that Methods 2 and 3 both perform well, but that Method 3 is slightly better.

Overall, we recommend Method 3, inverse probability weighting combined with multiple imputation of missing data among individuals eligible for sputum examination, as the method that provides the safest approach and the single best estimate of population pulmonary TB prevalence.

## Abbreviations

CC: Complete case; CI: Confidence interval; IPW: Inverse probability weighting; MAR: Missing at random; MCAR: Missing completely at random; MI: Multiple imputation; MNAR: Missing not at random; NTP: National TB Programme; PPS: Probability proportional to size; TB: Tuberculosis; WHO: World Health Organization.

## Competing interests

All authors have no competing interests to declare.

## Authors’ contributions

SF, CS, and KF wrote the paper; all co-authors suggested edits and gave comments on drafts of the manuscript, and all approved the final version. SF and CS led the analytical work, with important contributions from NY, RD, FM, PG, IO, and KF. RD, EB, ET, and FM contributed to the chapter in the WHO handbook on the analysis of TB prevalence surveys (2011). IO is the lead person in WHO for TB prevalence surveys. JL and RV took key roles in the planning and implementation of the TB prevalence survey that was conducted in the Philippines during 2007.

## Supplementary Material

Additional file 1 Multiple missing value imputation for analysis of pulmonary TB prevalence. Click here for file

## References

[B1] World Health Organization Global Tuberculosis Report 2012 Geneva: World Health Organization

[B2] World Health Organization Global Task Force on TB Impact Measurement Available from: http://www.who.int/tb/advisory_bodies/impact_measurement_taskforce/en/

[B3] World Health Organization Global Task Force on TB Impact Measurement TB impact measurement: policy and recommendations for how to assess the epidemiological burden of TB and the impact of TB control (Stop TB policy paper no. 2) 2009 Geneva: World Health Organization

[B4] World Health Organization Tuberculosis prevalence surveys: a handbook 2011 Geneva: World Health Organization

[B5] World Health Organization Assessing tuberculosis prevalence through population-based surveys 2007 Philippines: World Health Organization

[B6] Williams B Gopi PG Borgdorff MW Yamada N Dye C The design effect and cluster samples: optimising tuberculosis prevalence surveys Int J Tuberc Lung Dis 2008 12 10 1110 1115 18812038

[B7] Sterne JA White IR Carlin JB Spratt M Royston P Kenward MG Wood AM Carpenter JR Multiple imputation for missing data in epidemiological and clinical research: potential and pitfalls BMJ 2009 338 b2393 10.1136/bmj.b2393 19564179 PMC2714692

[B8] Carpenter JR Analysis of partially observed datasets: putting methodology into practice 2010 London: Royal Statistical Society

[B9] Tupasi TE Radhakrishna S Chua JA Mangubat NV Guilatco R Galipot M Ramos G Quelapio MI Beltran G Legaspi J Vianzon RG Lagahid J Significant decline in the tuberculosis burden in the Philippines ten years after initiating DOTS Int J Tuberc Lung Dis 2009 13 10 1224 1230 19793426

[B10] Hoa NB Sy DN Nhung NV Tiemersma EW Borgdorff MW Cobelens FG National survey of tuberculosis prevalence in Viet Nam Bull World Health Organ 2010 88 4 273 280 10.2471/BLT.09.067801 20431791 PMC2855599

[B11] Ministry of Health National tuberculosis prevalence survey in Eritrea 2005 Eritrea: Ministry of Health

[B12] Soemantri S Senewe FP Tjandrarini DH Day R Basri C Manissero D Mehta F Dye C Three-fold reduction in the prevalence of tuberculosis over 25 years in Indonesia Int J Tuberc Lung Dis 2007 11 4 398 404 17394685

[B13] Government of Myanmar Report on national tuberculosis prevalence survey 2009–10 2012 Myanmar: Ministry of Health, Government of Myanmar

[B14] Federal Ministry of Health First Ethiopian national population-based tuberculosis prevalence survey 2012 Ethiopia: Ethiopian Health and Nutrition Research Institute

[B15] Ministry of Health Second national tuberculosis prevalence survey Cambodia, 2011 2011 Cambodia: National Tuberculosis Control Programme

[B16] White IR Royston P Wood AM Multiple imputation using chained equations: issues and guidance for practice Stat Med 2011 30 4 377 399 10.1002/sim.4067 21225900

[B17] Seaman SR White IR Copas AJ Li L Combining multiple imputation and inverse-probability weighting Biometrics 2012 68 1 129 137 10.1111/j.1541-0420.2011.01666.x 22050039 PMC3412287

[B18] World Health Organization Rapid implementation of the Xpert MTB/RIF diagnostic test: technical and operational '"How-to"; practical considerations 2011 Geneva: World Health Organization

[B19] Carpenter JR Kenward MG White IR Sensitivity analysis after multiple imputation under missing at random: a weighting approach Stat Methods Med Res 2007 16 3 259 275 10.1177/0962280206075303 17621471

[B20] StataCorp LP Stata Statistical Software: Release 11 2009 Texas: College Station

[B21] The R Foundation for Statistical Computing R project for statistical computing 2013

[B22] SAS Institute Inc SAS/STAT software 2010 Cary, NC: SAS Institute Inc

[B23] Carlin JB Galati JC Royston P A new framework for managing and analysing multiply imputed data in Stata Stata J 2008 8 1 49 67

[B24] Carpenter JR Kenward MG Vansteelandt S A comparison of multiple imputation and doubly robust estimation for analyses with missing data J R Stat Soc 2006 169 571 584 10.1111/j.1467-985X.2006.00407.x

[B25] RealCom Centre for Multilevel Modelling 2010 UK: University of Bristol

