# Supplementary material for: Analysis of tuberculosis prevalence surveys: new guidance on best-practice methods
Source: Emerg Themes Epidemiol. 2013 Sep 28;10:10. doi: 10.1186/1742-7622-10-10 (PMC3853155; doi:10.1186/1742-7622-10-10)
Supplement: Additional file 1 — Multiple missing value imputation for analysis of pulmonary TB prevalence. [file 1742-7622-10-10-S1.doc]

**Additional file 1.** **Multiple missing value imputation for analysis of pulmonary TB prevalence**

Multiple missing value imputation is done using regression models, using a procedure called “imputation by chained equations”. In the context of a TB prevalence survey, it is expected that age, sex, stratum, and cluster are known for all individuals. But there will be missing data on TB symptoms (yes or no, for each symptom in the symptom screening questionnaire), field and central chest X-ray reading (normal or abnormal), smear and culture results, and the primary outcome of pulmonary TB (yes or no).

The definition of a “bacteriologically-confirmed pulmonary TB case” that is applied in a prevalence survey synthesises the evidence across all of: (1) at least one (sometimes two) culture results (2) two (sometimes three) smear results (3) the central X-ray reading and (4) TB symptoms. Taking this into account, we recommend that “bacteriologically confirmed pulmonary TB case yes/no” should not be included in the imputation models. Instead, it is better to include a variable for each of the two sputum smear results, and a variable for each culture result. It is also important to use “passive imputation” to create a composite variable for smear positivity from the combination of the two smear results – for example, that an individual is “smear-positive” if at least one of the smear results is positive. When imputing missing smear results for sputum sample 1, sputum sample 2 is used as an explanatory variable, and vice versa. When imputing other variables, the composite variable on smear positivity yes/no is used instead. If two specimens are cultured, the same approach is taken as for smear results, and a composite variable for culture positivity is created. Once the imputed datasets are created, in each dataset the survey case definition can be applied to create two new variables (1) smear-positive pulmonary TB and (2) bacteriologically-confirmed pulmonary TB.

The imputation is implemented as follows:

1. “Starting values” are assigned to all the missing data. For each variable, these “starting values” are obtained from a random sample of the values from individuals for whom data are not missing. Thus the “starting values” are “borrowed at random” from available data.
2. For each variable with missing data, a model is then fitted with this particular variable as the outcome and other variables as explanatory variables. This is done sequentially, in order of the proportion of data that are missing and starting with the variables with the least missing data.
3. For example, suppose the first step is to impute values of cough with a duration of 2 weeks or more, yes or no. The imputation model will be a logistic regression model with cough (yes or no) as the outcome variable, and each of age group, sex, stratum, other TB symptoms, field and central chest X-ray reading, and smear and culture positivity as explanatory variables. Note that age should be grouped and included in the model as a categorical variable (because it is unlikely that the log odds of TB follows a linear trend with age), for example 15–24 years, 25–34 years, 35–44 years, 45–54 years, 55+ years. Age should be grouped in such a way that the risk of TB is similar within each age group, and the particular grouping used may vary by country. Note also that only individuals with the cough variable observed are used in this step – i.e. when fitting each univariable imputation model the “borrowed imputations” from (1) are used only for the explanatory (independent) variables for that imputation model. The “borrowed imputations” for the dependent variable (in the particular imputation model being fitted, with cough yes or no being the dependent variable for this model) are discarded.

   The fitted imputation model is then used to obtain a predicted probability that an individual has a cough of 2 weeks or more duration (yes or no). Taking into account the uncertainty about the estimate of this predicted probability, a value of 0 (cough = no) or 1 (cough = yes) is imputed for each individual for whom data on cough were not available.

   Next, the observed and newly imputed data on cough, combined with the observed and “starting values” on other variables, are used to predict a second variable (the one with the second least amount of missing data). This might be coughing blood, yes or no. Exactly the same procedure is followed as above. Then the same thing is done for field chest X-ray reading, normal or abnormal, again using a logistic regression model. After this, the central chest X-ray reading is imputed. Next, smear positivity, yes or no, is imputed for sputum sample 1, and then for sputum sample 2. Finally, a logistic regression model with culture-positivity (yes or no) as the outcome variable, and age group, sex, stratum, TB symptoms, field and central chest X-ray reading, and smear-positivity, as explanatory variables, will be fitted. If the ratio of the prevalence of smear-positivity and/or culture-positivity in women compared with men varies by age group (among individuals with complete data), it will be necessary to include an interaction between age group and sex in the imputation models for smear-positivity and culture-positivity.
4. After this process has been completed once for each variable with missing data, then a dataset has been created in which all missing values have been imputed using a regression model. These newly imputed values are then used as “starting values” for the next iteration of the process. It is necessary to cycle through this process at least ten and preferably twenty times, in order to obtain one reliable imputed dataset in which the imputed values are not dependent on the original “starting values”.
5. Then the process in (1) to (4) is repeated several times, in order to obtain several imputed datasets (thus “multiple” missing value imputation). It is recommended to create at least 20 imputed datasets (see main text for justification).

In Stata, a limitation of the *ice* command used to implement “imputation by chained equations” is that it does not allow for correlation among individuals in the same cluster. However, because TB symptoms and the field and central chest X-ray reading are included as variables in the imputation models, at least part of the correlation among individuals in the same cluster will be explained by the models. It is now possible to impute data in a way that allows for correlation among individuals in the same cluster, using the Realcom package, and to then import the imputed datasets into Stata for analysis.
